# Parasite Biomass-Related Inflammation, Endothelial Activation, Microvascular Dysfunction and Disease Severity in Vivax Malaria

**DOI:** 10.1371/journal.ppat.1004558

**Published:** 2015-01-08

**Authors:** Bridget E. Barber, Timothy William, Matthew J. Grigg, Uma Parameswaran, Kim A. Piera, Ric N. Price, Tsin W. Yeo, Nicholas M. Anstey

**Affiliations:** 1 Global and Tropical Health Division, Menzies School of Health Research and Charles Darwin University, Darwin, Northern Territory, Australia; 2 Department of Infectious Diseases, Queen Elizabeth Hospital, Sabah, Malaysia; 3 Infectious Diseases Society Sabah-Menzies School of Health Research Clinical Research Unit, Kota Kinabalu, Sabah, Malaysia; 4 Centre for Tropical Medicine, Nuffield Department of Clinical Medicine, University of Oxford, Oxford, United Kingdom; 5 Lee Kong Chian School of Medicine, Nanyang Technological University, Singapore; 6 Insitute of Infectious Disease and Epidemiology, Tan Tock Seng Hospital, Singapore; 7 Department of Infectious Diseases, Royal Darwin Hospital, Darwin, Northern Territory, Australia; McGill University, Canada

## Abstract

*Plasmodium vivax* can cause severe malaria, however its pathogenesis is poorly understood. In contrast to *P. falciparum*, circulating vivax parasitemia is low, with minimal apparent sequestration in endothelium-lined microvasculature, and pathogenesis thought unrelated to parasite biomass. However, the relationships between vivax disease-severity and total parasite biomass, endothelial autocrine activation and microvascular dysfunction are unknown. We measured circulating parasitemia and markers of total parasite biomass (plasma parasite lactate dehydrogenase [pLDH] and PvLDH) in adults with severe (n = 9) and non-severe (n = 53) vivax malaria, and examined relationships with disease-severity, endothelial activation, and microvascular function. Healthy controls and adults with non-severe and severe falciparum malaria were enrolled for comparison. Median peripheral parasitemia, PvLDH and pLDH were 2.4-fold, 3.7-fold and 6.9-fold higher in severe compared to non-severe vivax malaria (p = 0.02, p = 0.02 and p = 0.015, respectively), suggesting that, as in falciparum malaria, peripheral *P. vivax* parasitemia underestimates total parasite biomass, particularly in severe disease. *P. vivax* schizonts were under-represented in peripheral blood. Severe vivax malaria was associated with increased angiopoietin-2 and impaired microvascular reactivity. Peripheral vivax parasitemia correlated with endothelial activation (angiopoietin-2, von-Willebrand-Factor [VWF], E-selectin), whereas markers of total vivax biomass correlated only with systemic inflammation (IL-6, IL-10). Activity of the VWF-cleaving-protease, ADAMTS13, was deficient in proportion to endothelial activation, IL-6, thrombocytopenia and vivax disease-severity, and associated with impaired microvascular reactivity in severe disease. Impaired microvascular reactivity correlated with lactate in severe vivax malaria. Findings suggest that tissue accumulation of *P. vivax* may occur, with the hidden biomass greatest in severe disease and capable of mediating systemic inflammatory pathology. The lack of association between total parasite biomass and endothelial activation is consistent with accumulation in parts of the circulation devoid of endothelium. Endothelial activation, associated with circulating parasites, and systemic inflammation may contribute to pathology in vivax malaria, with microvascular dysfunction likely contributing to impaired tissue perfusion.

## Author Summary

How vivax parasites cause severe malaria is not known. In contrast to falciparum parasites, the number of vivax parasites circulating in peripheral blood is low, and there is thought to be little sequestration of parasitized red cells within endothelium-lined small blood vessels in vital organs. Total parasite burden (circulating plus hidden) and activation and dysfunction of the endothelial cells lining blood vessels all contribute to severe disease in falciparum malaria, but have not been evaluated in severe vivax malaria. We measured parasite lactate dehydrogenase (pLDH) and *P. vivax*-pLDH (PvLDH) as proxies of total parasite biomass and found that, as in falciparum malaria, the total biomass of vivax parasites is underestimated by counting parasites circulating in peripheral blood, suggesting a hidden burden of vivax parasites. Markers of total vivax biomass were strongly associated with illness-severity and inflammatory cytokines, suggesting that this hidden burden is capable of contributing to generalised inflammation and hence severe disease. Number of peripheral vivax parasites, but *not* total biomass, correlated with activation of endothelial cells, suggesting that the hidden vivax-infected red cells may accumulate in parts of organs without endothelium, such as the slow-circulation of the spleen or non-blood-vessel parts of the bone marrow. Severe vivax malaria was associated with increased endothelial activation and impaired microvascular function, suggesting that these processes also contribute to impaired blood flow and disease.

## Introduction

While *P. falciparum* accounts for a majority of severe and fatal malaria cases worldwide, *P. vivax* is a major cause of morbidity outside of Africa, causing an estimated 70–390 million malaria cases per year [1]. Although previously considered benign, *P. vivax* is now recognized as capable of causing severe and fatal disease [2], [3], [4], [5], [6], [7], [8], [9], [10]. Despite this, little is known about the pathogenesis of severe disease in vivax malaria.

In falciparum malaria, severe and fatal disease is characterised by cytoadherence of parasitized red blood cells (RBCs) to activated and dysfunctional endothelium, leading to parasite sequestration with microvascular obstruction [11], [12], [13]. As a result of sequestration the mature schizont stages of *P. falciparum* are rarely seen in peripheral blood [14]. Total parasite biomass is underestimated by circulating parasitemia quantitated by microscopy of peripheral blood, and is more accurately quantitated by plasma *P. falciparum* histidine rich protein-2 (HRP2). Pathogenesis and disease severity in falciparum malaria are both biomass-related: in contrast to peripheral parasitemia [15], [16], [17], plasma HRP2 is strongly and independently correlated with disease severity and mortality among both children [17], [18], [19], [20] and adults [21], [22].

Cytoadherence of infected RBCs to endothelial cells is 10-fold less in *P. vivax* infection than in falciparum malaria [23], and autopsy evidence for sequestration within the endothelium-lined microvasculature of vital-organs in vivax malaria is minimal [2], [4], [24]. This, together with the lower parasitemias resulting from preferential invasion of reticulocytes, is thought to account for the lower lethality of *P. vivax* [2]. The paucity of apparent *endothelial* microvascular sequestration in vivax malaria has led to the assumption that peripheral parasitemia reflects total parasite biomass. However, this assumption has been questioned [3], [25], [26], with more adhesive schizont-stages of *P. vivax* known to be under-represented in peripheral blood [26], [27]. Accumulation of *P. vivax-*infected RBCs in the spleen or bone marrow has been hypothesised [3], [25], [26] but not yet systematically investigated. In contrast to falciparum malaria, no study has evaluated the relationships between total parasite biomass, disease severity and systemic inflammation in vivax malaria. We propose that in vivax malaria, parasite lactate dehydrogenase (pLDH) and *P. vivax*-pLDH (PvLDH), produced by viable or recently killed parasites, may be used to estimate total parasite biomass. While plasma pLDH has been shown to demonstrate only moderate correlation with peripheral parasitemia in vivax malaria [28], a pLDH antigen capture enzyme-linked immunosorbent assay demonstrated a direct relationship between parasite production of pLDH and total *P. vivax* parasite concentration *ex vivo*, including all parasite stages, suggesting that pLDH reflects total *P. vivax* parasite biomass [29]. As with HRP2 in falciparum malaria [30], pLDH is produced to a greater extent by *P. vivax* schizonts and trophozoites than by ring-form parasites [31], and given the under-representation of mature *P. vivax* stages in peripheral blood [26], [27], pLDH may be a better marker of total parasite biomass and a better prognostic indicator than peripheral parasitemia.

In falciparum malaria, clinical severity and mortality are also independently associated with impaired microvascular function [13] and with increased angiopoietin-2 (Ang-2) [32], a key product of endothelial Weibel-Palade Bodies (WPB) and an autocrine mediator of endothelial activation [33]. Despite the apparent paucity of endothelial microvascular sequestration in vital organs, *P. vivax* has been associated with greater endothelial activation than *P. falciparum*, with Ang-2 concentrations higher in non-severe vivax compared to non-severe falciparum malaria [34]. However the relationships between clinical severity, microvascular dysfunction and endothelial WPB exocytosis have not been assessed in vivax malaria.

We tested the hypotheses that in vivax malaria, peripheral parasitemia would underestimate total parasite biomass, and that markers of total parasite biomass would be related to systemic inflammation and clinical severity. We also determined the relationship between vivax disease severity and endothelial activation and microvascular function, with patients with non-severe and severe falciparum malaria included for comparison. We found that total vivax biomass was underestimated by peripheral parasitemia, and was associated with both systemic inflammation and disease severity in vivax malaria. Severe vivax malaria was associated with increased endothelial activation and WPB exocytosis and with impaired microvascular function, comparable to that seen in severe falciparum malaria.

## Results

### Patients

A total of 192 malaria patients and 74 healthy controls were enrolled. Malaria patients included 62 with vivax malaria (53 non-severe, 9 severe) and 130 with falciparum malaria (109 non-severe, 21 severe). The clinical and epidemiological features of 43 patients with vivax malaria (including 7 with severe disease) and 122 patients with falciparum malaria (including 13 with severe disease) have been previously reported [35]. Baseline characteristics are shown in Table 1. Among the 9 patients with severe vivax malaria, severity criteria included hypotension (n = 6), respiratory distress (n = 1), jaundice (n = 2), metabolic acidosis (n = 1), abnormal bleeding (n = 1) and multiple convulsions (n = 1). Three patients had 2 severity criteria and 6 had one. Pre-antibiotic blood cultures were negative in 4 patients, positive for *Streptococcus pneumoniae* in one [35], and not done in 4. No deaths occurred from either species.

**Table 1:** Baseline characteristics of vivax and falciparum malaria patients and healthy controls.

		*P. vivax*			*P. falciparum*		
	Controls (n = 74*)	Non-severe (n = 53)	Severe (n = 9)	P value (sev vs. ns)	Non-severe (n = 109)	Severe (n = 21)	P value (sev vs. ns)
**Age, years**							
median (IQR)	35 (23–44)	24 (18–39)	39 (30–52)	0.168	25 (17–39)	33 (19–45)	0.148
range	14–69	13–61	14–79		13–78	13–60	
**Males, n (%)**	52 (70)	39 (74)	9 (100)	0.105	79 (72)	15 (71)	0.922
**Weight, mean (sd); kg**	58 (10)	56 (12)	58 (8)	0.584	56 (13)	66 (17)	0.002
**Current smoker, n (%)**	30 (41)	26 (49)	1 (11)	0.034	37 (34)	10 (48)	0.232
**Fever duration, days; median (IQR)**		5 (3–7)	4 (3–4)	0.135	5 (3–7)	7 (5–7)	0.014
**Systolic blood pressure, mean (sd); mmHg**	123 (15)	115 (16)	119 (18)	0.451	116 (16)	114 (17)	0.756
**Pulse rate, mean (sd); beats/min**	70 (12)	87 (19)	97 (14)	0.172	92 (18)	98 (16)	0.123
**Respiratory rate, mean (sd); breaths/min**	20 (3)	24 (5)	26 (4)	0.181	27 (6)	31 (7)	0.006
**Temperature, mean (sd); °C**	36.5 (0.4)	37.4 (1.2)	36.9 (0.5)	0.224	37.9 (1.1)	38.1 (1.4)	0.407
**Time from malaria treatment to enrolment, hours; median (IQR)**	NA	5 (0 –– 11)	10 (6–15)	0.126	5 (0–12)	4 (0–10)	0.541

*includes all healthy controls who had Near Infrared Spectroscopy and/or blood analysis performed

### Peripheral parasitemia, schizontemia, markers of total parasite biomass and disease severity

**Figure 1:**
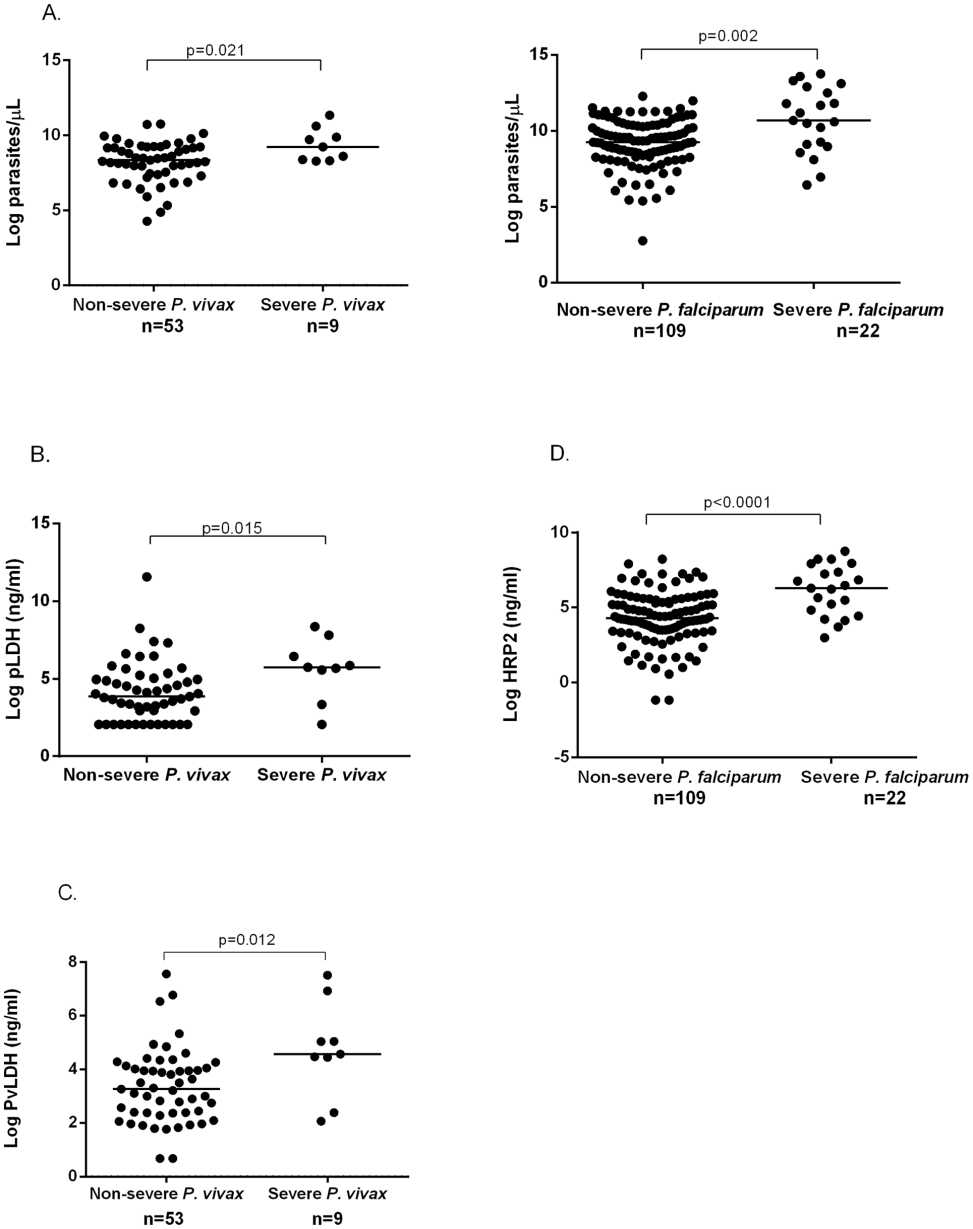
Parasitemia (A) and total parasite biomass [pLDH (B), PvLDH (C), and HRP2 (D)] among patients with severe and non-severe vivax and falciparum malaria.

In patients with vivax malaria, the median peripheral parasitemia was 2.4-fold higher in severe compared to non-severe disease (10,243 parasites/µL vs. 4,209 parasites/µL; P = 0.021), while the median PvLDH was 3.7-fold higher (96.6 ng/ml vs. 26.2 ng/ml; P = 0.021) and pLDH 6.9-fold higher (308 ng/ml vs. 44.6 ng/ml; P = 0.015) (Table 2 and Fig. 1). After removing the patient with severe vivax malaria and concurrent bacteremia from the analysis, the median peripheral parasitemia was 1.8-fold higher in severe compared to non-severe disease (7,865 parasites/µL vs. 4,209 parasites/µL; P = 0.047), while the median PvLDH was 4.8-fold higher (125 ng/ml vs. 26.2 ng/ml; P = 0.002) and pLDH 7.3-fold higher (326 ng/ml vs. 44.6 ng/ml; P = 0.002). In falciparum malaria, median peripheral parasitemia and plasma HRP2 were 4.2-fold and 7.4-fold higher, respectively, in severe compared to non-severe disease (Table 2).

**Table 2:** Parasite count and parasite biomass among patients with P. vivax and P. falciparum malaria.

	*P. vivax*			*P. falciparum*		
	Non-severe (n = 53)	Severe (n = 9)	P value (sev vs. ns Pv)	Non-severe (n = 109)	Severe (n = 21)	P value (sev vs. ns Pf)
**Parasite count (parasites/µL)**	4,209 (1700–10,165)	10,243 (4387–19,520)	0.021	10,500 (4014–32,267)	44,332 (9079–273, 909)	0.002
**PvLDH (ng/ml)**	26.2 (10.7–57.3)	96.6 (85.6–154)	0.012			
**PvLDH/Log parasitemia**	3.06 (1.33–7.73)	10.42 (9.27–18.60)	0.027			
**pLDH (ng/ml)**	44.6 (7.8–142)	308 (258–619)	0.015	50.23 (22.9–121)	86.79 (39.3–452)	0.016
**pLDH/Log parasitemia**	6.46 (1.60–20.1)	36.7 (27.9–58.3)	0.021	5.54 (2.33–13.13)	7.58 (3.51–65.65)	0.058
**HRP2 (ng/ml)**				72.8 (32.8–260)	541 (125–1590)	<0.0001
**HRP2/Log parasitemia**				9.04 (3.38–23.55)	49.0 (13.8–196)	0.0002

Note: Values are median (inter-quartile range).

Ratios are calculated as ngs/1000 peripheral parasites, with the denominator log-transformed to a normal distribution.

To estimate whether the “hidden parasite biomass” was larger in severe compared to non-severe malaria, we calculated the ratio of plasma pLDH and plasma PvLDH concentration to peripheral parasite density in vivax malaria, and the ratio of plasma HRP2 concentration to peripheral parasite density in falciparum malaria. Among patients with vivax malaria the ratios of PvLDH and pLDH to parasite density (log) were 3.7-fold and 5.7-fold higher, respectively, in severe compared to non-severe disease. The corresponding fold-increase in the ratio of plasma HRP2 to parasite count (log) in patients with severe compared to non-severe falciparum malaria was 5.4.

**Figure 2:**
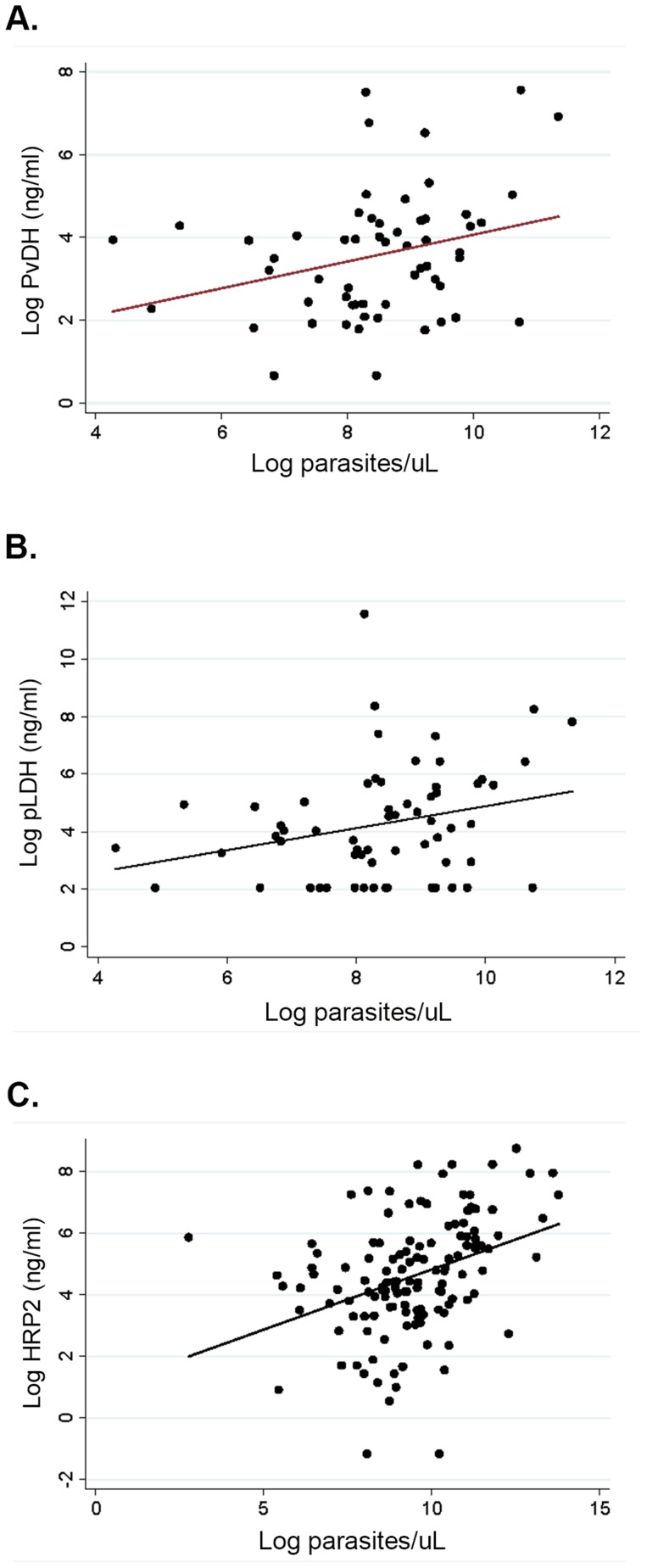
Parasitemia and markers of total parasite biomass among patients with vivax and falciparum malaria. (**A**) Correlation between parasitemia and PvLDH among patients with vivax malaria, Spearman's ρ = 0.27, P = 0.033, (**B**) Correlation between parasitemia and pLDH among patients with vivax malaria, Spearman's ρ = 0.27, P = 0.028, and (**C**) correlation between parasitemia and HRP2 among patients with falciparum malaria, Spearman's ρ = 0.41, P = <0.0001.

Among patients with vivax malaria pLDH and PvLDH were strongly correlated (ρ = 0.90, P<0.0001). However there was only a modest correlation between PvLDH and parasitemia (ρ = 0.27, P = 0.033), and between pLDH and parasitemia (ρ = 0.28, P = 0.028), comparable to the correlation between parasitemia and plasma HRP2 among patients with falciparum malaria (ρ  = 0.41, P<0.0001) (Fig. 2).

Among patients with vivax malaria who had detectable pLDH and PvLDH levels on enrolment and in whom longitudinal measurements could be performed (until day 3 or until undetectable), levels were undetectable by day 3 in 16/25 (64%) and 22/31 (71%) patients respectively.

In *ex vivo* assay conditions, *P. vivax* parasites exist as mature schizonts for an estimated 5% of their 48-hour life-cycle [36]. Despite this, only 14/59 (24%) patients with vivax malaria had any circulating schizonts detectable on peripheral blood film, including 2/9 (22%) with severe and 12/50 (24%) with non-severe disease. Of those with schizonts detectable, schizonts comprised <5% of circulating parasites in 11/14 (78%) patients, including one with severe and 10 with non-severe disease. There was a wide variation in the proportion of peripheral *P. vivax* parasites at ring (median 33%, range 0–100%, IQR 9–77%) and trophozoite stage (median 67%, range 0–100%, IQR 20–91%). Among patients with severe and non-severe vivax malaria, there were no differences between the median percentage of rings or trophozoites in peripheral blood.

### Markers of endothelial activation and thrombosis, cytokines, and clinical disease

Median concentrations of Ang-2 and von Willebrand Factor (vWF), the two major products of endothelial WPB release, were both increased in patients with severe and non-severe vivax malaria compared to controls (P<0.01 for all comparisons), with Ang-2 nearly twice as high in severe compared to non-severe disease (P<0.0001) (Table 3). The median concentrations of the endothelial adhesion receptors E-selectin and ICAM1 were also increased in severe and non-severe vivax malaria compared to controls (P<0.001 for all comparisons). In patients with vivax malaria there was no association between age and any of the endothelial activation markers measured. Median concentrations of Ang-2, E-selectin and ICAM1 were at least as high in severe and non-severe vivax malaria as they were in severe and non-severe falciparum malaria (Table 3).

**Table 3:** Laboratory and physiological measurements among patients with vivax and falciparum malaria and healthy controls.

		*P. vivax*			*P. falciparum*		
	Controls	Non-severe (n = 53)	Severe (n = 9)	P value (sev vs. ns Pv)	Non-severe (n = 109)	Severe (n = 21)	P value (sev vs. ns Pf)
Haematology							
Platelets, × 10^3^/µL		70 (56–105)	29 (18–71)	0.021	71 (40–111)	38 (22–63)	0.001
Platelet nadir, 10^3^/µL		66 (49–61)	29 (18–62)	0.022	59 (37–89)	31 (20–54)	0.001
Neutrophils, × 10^3^/µL		3.25 (2.70–4.60)	3.24 (2.16–4.41)	0.992	3.1 (2.4–4.5)	3.5 (2.6–4.6)	0.952
Haemoglobin, g/dL		12.4 (11.4–13.8)	14.5 (12.7–14.7)	0.022	13.2 (11.8–14.3)	13.1 (11.4–14.1)	0.606
Haemoglobin nadir, g/dL, mean (SD)		11.4 (2.01)	11.4 (0.8)	0.907	11.6 (1.56)	10.1 (2.04)	0.002
Markers of endothelial activation							
Ang-2†, pg/ml	1183 (875–1597)	4557 (3463–6197)	8857 (6547–9743)	0.001	3230 (2123–5243)	8371 (3963–13,463)	<0.0001
VWF*, pg/ml	1156 (843–1634)	4498 (3108–5252)	4137 (3142–5561)	0.986	5106 (3906–7111)	5873 (4552–7424)	0.537
ICAM1†, pg/ml	149 (123–167)	505 (416–639)	686 (682–832)	0.077	504 (388–660)	660 (538–789)	0.007
E-selectin†, pg/ml	19.3 (13.0–24.9)	68.2 (42.6–93.4)	72.8 (65.1–115.4)	0.542	55.5 (37.2–80.1)	45.6 (31.9–75.8)	0.477
Markers of microvascular thrombosis							
ADAMTS13*, ng/ml	655 (594–726)	513 (399–588)	505 (381–599)	0.797	526 (419–573)	331 (242–385)	0.0001
ADAMTS13 Activity ng/ml*	686 (601–762)	556 (434–629)	419 (362–516)	0.027	419 (346–507)	236 (195–288)	<0.0001
Markers of systemic inflammation							
IL-6∧, pg/ml	BDL (27/30)	27.6 (9.13–93.6)	49.52 (36.1–54.0)	0.369	46.4 (18.5–89.7)	112 (42.0–357)	0.001
IL-10∧, pg/ml	BDL (29/30)	186 (41.3–504)	173 (106–319)	0.737	180 (85.4–272)	464 (211–1135)	0.001
Microvascular perfusion							
Lactate, mmol/L		1.17 (0.8–1.49)	1.28 (1–2.23)	0.197	1.22 (0.91–1.62)	2.03 (1.03–2.6)	0.007
Microvascular reactivity# ^,^ Units/second	6.34 (5.21–7.03)	6.98 (5.79–7.52)	5.44 (3.62–6.01)	0.006	6.31 (4.87–7.43)	4.88 (3.70–6.74)	0.024

Investigations are performed on enrolment, unless otherwise stated. Numbers are median (IQR) unless otherwise stated. Where a result is below the lower limit of detection (LLD), half the LLD is substituted.

For intergroup comparison between controls, non-severe *P. vivax* and severe *P. vivax*; and between controls, non-severe *P. falciparum* and severe *P. falciparum;* P values were <0.05 (by Kruskal-Wallis or anova) for all variables tested. All significant differences in laboratory measurements between patients with severe and non-severe vivax malaria remain significant after excluding a severe malaria patient with concurrent pneumococcal bacteremia.

† measured in 50 healthy controls and all patients with malaria

* measured in 30 healthy controls, all patients with severe malaria, 29 patients with non-severe *P. vivax* and 35 patients with non-severe *P. falciparum*

∧ measured in 30 healthy controls, all patients with severe malaria, all patients with *P. falciparum*, and 46 patients with non-severe *P. vivax*

# measured in 67 controls, 8 patients with severe *P. vivax*, 33 patients with non-severe *P. vivax*, 19 patients with severe *P. falciparum*, and 72 patients with non-severe *P. falciparum*

The median concentrations of the vWF-cleaving protease, ADAMTS13, and its activity, were lower among patients with severe and non-severe vivax malaria compared to controls (P<0.001 for all comparisons), with median ADAMTS13 activity also lower in severe compared to non-severe vivax malaria (P = 0.027).

Median concentrations of IL-6 and IL-10 were higher in severe and non-severe vivax malaria compared to controls (Table 3).

### Parasitemia, markers of total parasite biomass, and biomarkers of severity in vivax and falciparum malaria

In patients with vivax malaria, peripheral parasitemia correlated with the endothelial WPB products, VWF (ρ  = 0.53, P<0.0001) and Ang-2 (ρ = 0.36, P = 0.004), as well as the endothelial adhesion receptor E-selectin (ρ = 0.33, P = 0.009), and was inversely correlated with activity of the VWF-cleaving protease ADAMTS13 (ρ = −0.31, P = 0.055) (Table 4). Importantly, and in contrast to peripheral parasitemia, no correlation was seen between either of the total parasite biomass markers PvLDH or pLDH, and the endothelial products VWF, Ang-2, or E-selectin (Table 4).

**Table 4:** Correlation coefficients of baseline parasitemia and parasite biomass markers among patients with vivax and falciparum malaria.

		*P. vivax*					*P. falciparum*			
	n	parasitemia	P value	PvLDH	P value	n	parasitemia	P value	HRP2	P value
Platelets	62	−0.179	0.163	−0.256	0.045	130	−0.323	0.0002	−0.326	0.0001
Platelet nadir	62	−0.185	0.151	−0.268	0.035	130	−0.295	0.0006	−0.234	0.008
Neutrophils	62	0.145	0.262	−0.077	0.554	130	0.221	0.012	0.057	0.525
Haemoglobin	62	0.151	0.242	0.209	0.103	130	0.124	0.159	−0.218	0.014
Haemoglobin nadir	62	−0.059	0.648	0.071	0.583	130	−0.052	0.555	−0.375	<0.0001
Ang-2	62	0.362	0.004	0.134	0.299	130	0.447	<0.0001	0.500	<0.0001
VWF	38	0.525	0.0007	0.229	0.167	56	0.289	0.031	0.239	0.079
E-selectin	62	0.329	0.009	0.096	0.456	130	0.196	0.026	−0.038	0.668
ICAM1	62	0.227	0.076	0.026	0.839	130	0.138	0.119	0.220	0.013
ADAMTS13	38	−0.092	0.584	0.034	0.840	56	−0.057	0.679	−0.417	0.002
ADAMTS13 Activity	38	−0.314	0.055	−0.083	0.620	56	−0.256	0.057	−0.517	0.0001
IL-6	55	0.308	0.022	0.271	0.045	130	0.534	<0.0001	0.331	0.0001
IL-10	55	0.233	0.087	0.284	0.035	130	0.417	<0.0001	0.341	0.0001

Note: Among patients with vivax malaria there were no significant correlations between pLDH and other variables.

All significant associations remain significant after excluding a patient with severe vivax malaria and concurrent pneumococcal bacteremia.

While not associated with markers of endothelial activation, PvLDH was correlated with the systemic inflammatory markers IL-6 (ρ = 0.27, P = 0.045) and IL-10 (ρ = 0.28, P = 0.035), and, in contrast to peripheral parasitemia, was inversely correlated with baseline platelet count (ρ = −0.26, P = 0.045) and with platelet nadir (ρ = −0.27, P = 0.035).

Among patients with falciparum malaria, biomarkers of systemic inflammation correlated with both peripheral parasitemia and the total parasite biomass marker, plasma HRP2 (Table 4). In addition, and in contrast to vivax malaria, endothelial activation correlated not only with peripheral parasitemia but also with total parasite biomass (Table 4). Baseline platelet count and platelet nadir also correlated with both peripheral parasitemia and HRP2 (Table 4).

### Ang-2, ADAMTS13, and biomarkers of severity among patients with vivax malaria

In patients with vivax malaria, Ang-2, in addition to correlating with parasitemia, correlated with the adhesion receptors E-selectin (ρ = 0.40, P = 0.001) and ICAM-1 (ρ = 0.54, P<0.0001), and inversely with

ADAMTS13 antigen (ρ = −0.41, P = 0.010) and activity levels (ρ = −0.60, P = 0.0001), with all associations remaining significant after adjusting for parasitemia.

ADAMTS13 antigen and activity levels correlated with platelet count (ρ = 0.36, P = 0.025; and ρ = 0.56, P = 0.0003, respectively), with this association remaining significant after adjusting for parasitemia. IL-6, elevated in vivax malaria, is known to be a major inhibitor of ADAMTS13 activity [37]. In vivax malaria, IL-6 was inversely correlated with ADAMTS13 activity (ρ = −0.40, P = 0.013) and platelet nadir (r = −0.44, p = 0.0009). No association occurred between lactate and ADMATS13 antigen and activity levels, or between lactate and Ang-2.

### Microvascular function

**Figure 3:**
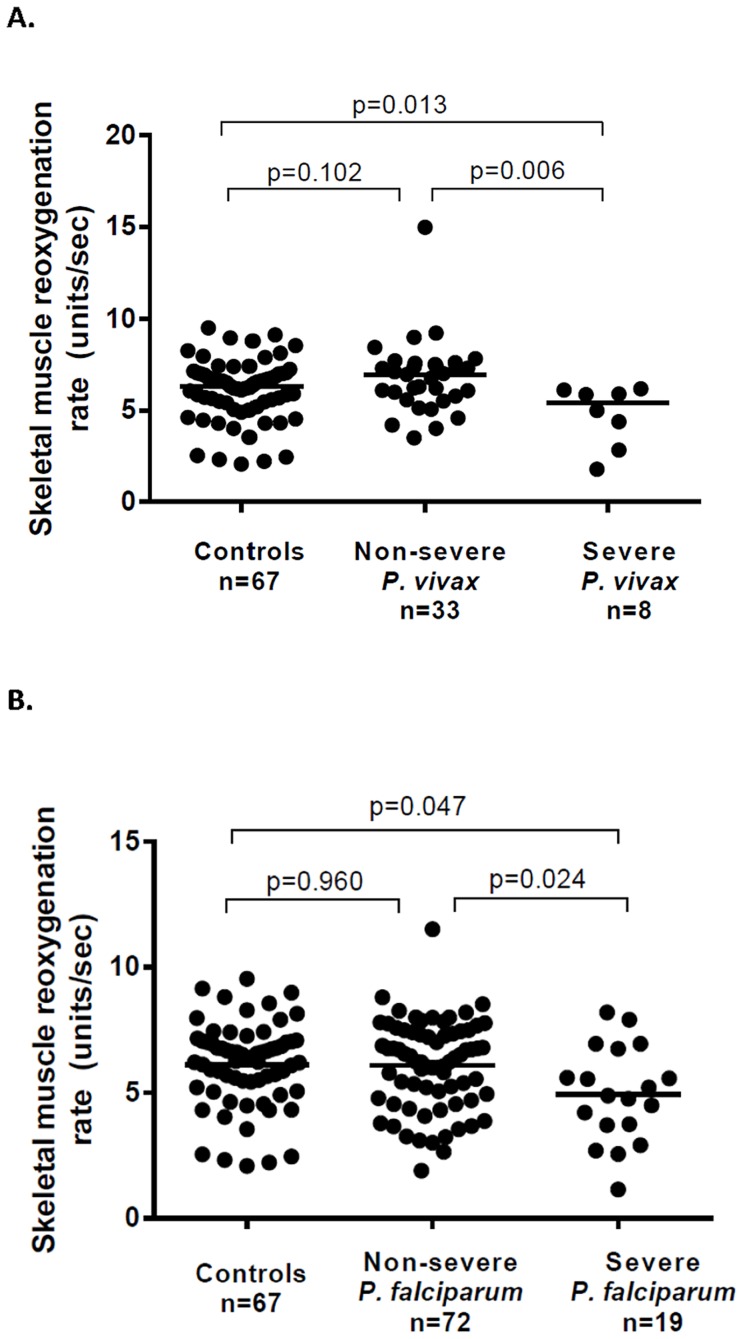
Microvascular function (skeletal muscle reoxygenation) among patients with vivax (A) and falciparum (B) malaria.

Microvascular reactivity (assessed using Near Infrared Resonance Spectroscopy [13]) was decreased in patients with severe vivax malaria (median 5.44 units/sec) compared to those with non-severe vivax malaria (median 6.98 units/sec; P = 0.006) and to controls (median 6.34, P = 0.027) (Table 3 and Fig. 3). Microvascular reactivity was also impaired among patients with severe falciparum malaria (median 4.88 units/sec) compared to those with non-severe falciparum malaria (6.31 units/sec; P = 0.024) and controls (P = 0.016), with the degree of impairment similar between severe vivax and severe falciparum malaria. In both species, there was no significant difference between controls and patients with non-severe malaria.

Among patients with vivax malaria, microvascular reactivity was inversely associated with the perfusion marker, lactate, in severe (ρ = −0.74, P = 0.04), but not non-severe disease (ρ = 0.09, P = 0.62). In severe disease ADAMTS13 antigen and activity levels correlated with microvascular reactivity (ρ = 0.64 and P = 0.09, for both associations), although no association occurred in non-severe disease (ρ = 0.07, P = 0.78; and ρ = −0.02, P = 0.95; respectively). No association was found between microvascular reactivity and other variables in vivax malaria, including parasitemia or biomass markers.

In patients with falciparum malaria, microvascular reactivity was inversely correlated with parasitemia (ρ = −0.22, P = 0.035), and positively correlated with platelet count (ρ = 0.017, P = 0.017). Among patients with severe and non-severe falciparum malaria, no association occurred between microvascular reactivity and lactate, or between microvascular reactivity and ADAMTS13 antigen or activity. In both species there was no association between microvascular reactivity and age.

Among patients with vivax malaria, 6 patients with severe and 16 with non-severe disease had NIRS repeated on day 3, with no change from baseline noted in either group (median microvascular reactivity on day 3  =  5.59 units/sec and 7.39 units/sec respectively). In severe falciparum, microvascular reactivity improved from 4.88 units/sec at enrolment to 6.51 units/sec on day 3 (P = 0.039), although no difference was noted among 37 patients with non-severe falciparum malaria (median microvascular reactivity on day 3 = 6.56 units/sec, P = 0.437)

## Discussion

Peripheral parasitemia underestimates measures of total parasite biomass in vivax malaria and circulating schizonts are under-represented in peripheral blood, suggesting tissue accumulation of a hidden biomass of *P. vivax*-infected RBCs. The proportions of total parasite biomass detectable in peripheral blood in uncomplicated and severe disease were of comparable magnitude in vivax and in falciparum malaria. The association between *P. vivax* peripheral parasitemia and total parasite biomass is modest, with total parasite biomass more strongly associated with disease severity compared to peripheral parasitemia. The smaller proportion of total *P. vivax* biomass detectable in peripheral blood in severe compared to non-severe disease suggests that hidden biomass is greater in severe vivax malaria and contributes to pathogenesis of disease. The association of total vivax parasite biomass with systemic inflammation suggests that the hidden biomass is capable of mediating systemic inflammatory pathology. In contrast to peripheral *P. vivax* parasitemia, total parasite biomass did not correlate with endothelial activation, and we therefore speculate that accumulation of *P. vivax*-infected RBCs may occur in parts of the circulation devoid of endothelium.

In falciparum malaria, peripheral parasitemia underestimates the total parasite biomass due to sequestration of parasitized RBCs within endothelium-lined microvasculature. While *P. vivax* is not thought to sequester in the endothelium-lined microvasculature to the same degree as *P. falciparum*, limited histopathological reports show intact *P. vivax*-infected RBCs in the bone marrow [38], [39], [40], [41], [42], [43] and spleen [44], [45], organs containing circulatory compartments that are not endothelium-lined. The spleen is a lymphoid organ whose primary role is to clear abnormal erythrocytes from the circulation, and hence plays a fundamental role in removing parasitized RBCs, especially in falciparum malaria where RBC deformability is markedly decreased. In falciparum malaria, endothelial cytoadherence of infected RBCs enable the parasite to sequester in non-splenic microvasculature and avoid passage through the spleen. In contrast, *P. vivax*-infected RBCs cytoadhere with less avidity to endothelial cells [23], and demonstrate increased deformability [46], [47], allowing circulation through the spleen. While 80-90% of splenic blood flow occurs through endothelial-lined sinuses, the remainder circulates through a parallel slow, open circulation in the splenic cords, devoid of endothelial cells [48]. In a recent report involving a splenectomised patient with vivax malaria, large numbers of intact non-phagocytosed *P. vivax*-infected reticulocytes were found in the splenic cords [44]. Accumulation of *P. vivax*-infected reticulocytes in the slow open microcirculation of the spleen, possibly through reticulocyte adherence to non-endothelial resident cells [49], [50], [51], [52], has been proposed as a mechanism by which *P. vivax* may avoid macrophage clearance [53] yet readily invade new reticulocytes.

The findings of our study suggest that tissue accumulation of *P. vivax* does occur and is greatest in severe vivax malaria, and we speculate it may occur in a location devoid of endothelium, such as the slow circulation of the spleen. We found no correlation between the *P. vivax* biomarkers of total biomass, pLDH and PvLDH, and either of the two products of endothelial WPB release, Ang-2 or VWF, or the endothelial adhesion receptor E-selectin. In contrast, peripheral parasitemia correlated with endothelial activation (Ang-2, VWF and E-selectin) in both vivax and falciparum malaria, and with total biomass (HRP2) in falciparum malaria. These findings suggest that endothelial cells may be activated by *P. vivax*-infected RBCs circulating through the peripheral microvasculature, but not by the hidden *P. vivax* biomass. Accumulation of *P. vivax* in the endothelium-free open circulation of the spleen may account for these findings. The finding that *P. vivax* peripheral parasitemia and markers of total parasite biomass both correlated with the leukocyte-derived cytokines may reflect the ability of circulating and tissue-accumulated *P. vivax* to stimulate leukocytes in both peripheral blood and leukocyte-containing organs such as the spleen or bone marrow, and thereby mediating organ dysfunction secondary to systemic inflammation.

Although underestimating total biomass, peripheral parasitemia was still higher in severe compared to non-severe malaria in both *P. vivax* and *P. falciparum*, and endothelial activation was higher in patients with severe disease. Ang-2 concentrations were twice as high in severe compared to non-severe vivax malaria, and comparable to levels seen in severe falciparum malaria. Ang-2, which causes autocrine endothelial activation, has previously been shown to be markedly elevated in severe falciparum malaria and a consistent predictor of death in both adults [32], [54], [55] and children [56], [57]. In severe falciparum malaria Ang-2 correlates with impaired endothelial function, lactate, plasma HRP2, ICAM-1, and E-selectin [32]. In our study, Ang-2 was also associated with ICAM-1 and E-selectin among patients with vivax malaria, consistent with the role of Ang-2 in activating endothelium in vivax as well as falciparum malaria [32]. Our findings of increased endothelial activation in severe vivax malaria are consistent with a recent study reporting higher concentrations of ICAM-1 and VCAM-1 in severe compared to uncomplicated vivax malaria [58].

Microvascular function was significantly impaired in severe vivax malaria, comparable to the impairment seen in severe falciparum malaria. As previously shown in severe falciparum malaria [13], the impairment of microvascular reactivity in severe vivax malaria was strongly associated with blood lactate, suggesting that impaired tissue perfusion is contributing to organ dysfunction in severe vivax malaria.

Plasma ADAMTS13 activity was decreased in vivax malaria, with deficiency associated with both severe disease and with impaired microvascular function. ADAMTS13 is a protease that cleaves ultra-large and prothrombogenic VWF multimers (UL-VWF), and deficiency leads to accumulation of UL-VWF with resultant increase in platelet aggregation and adhesion, and microvascular thrombosis. Accumulation of UL-VWF resulting from ADAMTS13 deficiency is characteristic of the microangiopathic disease thrombotic thrombocytopenic purpura (TTP), and has been reported in patients with both falciparum and vivax malaria [59]. While renal failure was not a feature of patients with severe vivax malaria in our study, thrombotic microangiopathy has been reported in severe vivax disease elsewhere [60], [61]. In our study, lower ADAMTS13 antigen and activity levels were associated with lower platelet counts, and with increased concentrations of IL-6, a known specific inhibitor of ADAMTS13 activity [37]. We speculate that biomass-related IL-6 may contribute to impaired ADAMTS13 activity and accumulation of UL-VWF multimers, and may thereby contribute to microvascular dysfunction, thrombocytopenia and disease severity in vivax malaria. Further studies however are required to confirm the mechanisms underlying ADAMTS13 deficiency in vivax malaria, and to investigate the role of thrombotic microangiopathy.

Our study had several limitations. Firstly, pLDH and PvLDH have not been previously validated as biomass markers in vivax malaria. Other factors may contribute to the plasma concentrations of these markers, such as host metabolic clearance rate, distribution within the host and diffusion rates from host tissue, and natural variation in parasite expression. Release of pLDH solely at the time of schizont rupture may provide a possible alternative explanation for the association between concentrations of pLDH/PvLDH, systemic inflammation and disease severity, with schizogony also associated with an inflammatory response [62]. However, we have demonstrated that *ex-vivo* concentrations of PvLDH increase progressively during short term culture of *P. vivax*, indicating that PvLDH is secreted throughout the parasite life-cycle and supporting the use of pLDHand PvLDH as surrogate markers of parasite biomass. Host inflammatory response may contribute in part to the marked under-representation of *P. vivax* schizonts in peripheral blood, however this would apply similarly to hosts infected with other *Plasmodium* species with under-representation of schizonts in peripheral blood, such as *P. falciparum* or *P. coatneyi*, whose schizonts have been clearly shown to sequester in tissues [63].

The small number of patients with severe vivax malaria (n = 9) limited our ability to evaluate the association between disease severity and other variables, and to assess for associations of parasite biomass and/or parasitemia that may have occurred only among patients with severe disease. In addition, with multiple comparisons we are unable to exclude the possibility that associations may have occurred by chance. However, the magnitude and direction of the associations with disease severity and with different measures of biomass are consistent, plausible, and are comparable to those in patients with falciparum malaria. Pre-antibiotic blood cultures were not performed in all our patients, and hence it is possible that as in falciparum malaria [64], [65], concurrent bacteremia may have contributed to the clinical features of some patients with severe vivax malaria. However, the much higher biomass in patients with severe compared to non-severe vivax cannot have been accounted for by concurrent bacteremia, or the parasitemia- or biomass-related associations we found. Finally, while we demonstrated increased endothelial activation among patients with vivax malaria in proportion to disease severity [34], the causes of WPB exocytosis in vivax malaria were not evaluated and require further study.

In summary, peripheral parasitemia underestimates total parasite biomass in vivax malaria, particularly in severe disease, suggesting tissue accumulation of a hidden biomass of *P. vivax*-infected RBCs. While histological studies are needed to confirm this, we propose that a hidden *P. vivax* biomass is capable of mediating systemic inflammation which may contribute to organ dysfunction. The correlation of markers of total *P. vivax* biomass with systemic inflammation but not with markers of endothelial activation is consistent with the hypothesis that accumulation of *P. vivax-*infected red cells may occur in parts of the circulation devoid of endothelium, such as the open circulation of the spleen or extravascular bone marrow. In contrast only peripheral parasitemia was associated with endothelial activation and the Weibel Palade Body products Ang-2 and VWF. The association between clinical severity and endothelial activation, ADAMTS13 deficiency, thrombocytopenia, and impaired microvascular function suggests that thrombotic microangiopathy, systemic inflammation and microvascular dysfunction may contribute to pathogenesis of disease in vivax malaria.

## Methods

### Study site and patients

This study was conducted in Sabah, Malaysia, a region currently in the pre-elimination phase of malaria control and where *P. vivax* endemicity is low [66], [67]. Patients were enrolled as part of a prospective clinical and epidemiological study of all malaria patients admitted to Queen Elizabeth Hospital, an adult tertiary referral hospital [35]. Consecutive patients with PCR-confirmed vivax or falciparum monoinfection were enrolled from September 2010 – October 2012 (with non-severe falciparum malaria patients included up until October 2011) if they were non-pregnant, ≥12 years old, had no major comorbidities or concurrent illness, were within 18 hours of commencing antimalarial treatment, had haemoglobin >7.0 g/dL, and had not been previously enrolled in the study. Clinical details of patients enrolled from September 2010 – October 2011, in addition to details regarding excluded patients, have been previously reported [35]. Severe malaria was defined as the presence of ≥1 of: unrousable coma (Glasgow Coma Scale score <11); multiple ( >2) convulsions; respiratory distress (respiratory rate >30 breaths per minute and oxygen saturation <94%); hypotension (systolic blood pressure ≤80 mm Hg); jaundice (bilirubin >43 µmol/L plus parasitemia >20 000/µL [*P. vivax*] or >100,000 [*P. falciparum*] and/or creatinine >132 µmol/L); significant abnormal bleeding; hypoglycaemia (blood glucose <2.2 mmol/L); metabolic acidosis (bicarbonate <15 mmol/L or lactate >4 mmol/L); acute kidney injury (AKI; creatinine >265 µmol/L); hyperparasitemia (*P. falciparum* parasitemia >10%). Healthy controls were visitors or relatives of malaria patients, with no history of fever in the past 48 hours and with blood film negative for malaria parasites.

Standardized history and physical examination were documented. Haematology, biochemistry, acid-base parameters, and lactate (by bedside blood analysis; iSTAT system) were obtained on admission. Parasite counts were determined by microscopy, and parasite species were identified by PCR [68], [69]. Patients were treated according to hospital guidelines, as previously described [35].

### Endothelial activation, WPB release, ADAMTS13 and cytokines

Venous blood collection in lithium heparin was centrifuged within 30 minutes of collection and plasma was stored at −70°C. Plasma concentrations of the endothelial activation markers ICAM-1, E-selectin, and Ang-2 were measured using ELISA (R&D Systems), and IL-6 and IL-10 were measured by flow cytometry (BD Cytometric Bead Array). Antigen concentrations of vWF and the vWF-cleaving enzyme, ADAMTS13, were measured in platelet poor plasma using ELISA (America Diagnostica), and ADAMTS 13 activity by fluorescence resonance energy transfer (FRET), as previously described [70].

### Parasitemia and markers of total parasite biomass

Peripheral blood parasitemia was quantitated per 200 white blood cells by microscopy and expressed as parasites/µL based on automated white cell count. Parasite stage distribution was quantitated on microscopy of peripheral blood. Plasma HRP2 (for *P. falciparum*) [32], genus-specific pLDH (for *P. falciparum* and *P. vivax*) and PvLDH (for *P. vivax*) were measured by ELISA [28] as proxies for total parasite biomass. Ratios of plasma pLDH and PvLDH to peripheral parasite density were expressed as ngs/1000 peripheral parasites, with the denominator log-transformed.

### PvLDH production *in vitro*

To confirm evidence of PvLDH secretion across the parasite life-cycle, four cryopreserved *P. vivax* isolates with a high proportion of ring-stages were thawed and cultured *ex-vivo* as previously described [71], over 48–54 hours. Starting parasitemias were 3623/µL (98% rings), 10,605/µL (98% rings), 19,342/µL (65% rings), and 24,680/µL (98% rings). Thick and thin films were prepared at serial time points for stage differential and culture supernatant sampled for concentration of PvLDH. A progressive increase in concentration of PvLDH in culture supernatant across the parasite life-cycle was demonstrated (**Text S1**), indicating secretion of PvLDH by all parasite stages. *In vitro* parasitemia remained stable throughout the culture duration.

### Microvascular function

Microvascular function was assessed using Near Infrared Spectroscopy (InSpectra 650, Hutchinson Technology, Hutchinson, MN) which uses a probe applied to the thenar eminence to noninvasively measure microcirculatory oxygenation (tissue oxygen saturation; StO_2_) before and after an ischaemic stress, as previously described [13]. To induce an ischaemic stress, a vascular cuff was inflated to 200 mm Hg for 5 minutes and then rapidly deflated. Microvascular function was defined as the gradient of the StO_2_ recovery slope from release of the vascular cuff until StO_2_ had reached 85% of the baseline value.

### Statistical methods

Statistical analysis was performed with STATA software (version 10.1; Statacorp, College Station, TX, USA). For continuous variables intergroup differences were initially compared using analysis of variance or Kruskal-Wallis tests depending on distribution. Student's T-test or Mann-Whitney tests were used for post-hoc pair-wise comparisons. Categorical variables were compared using χ^2^ or Fisher's exact test. Associations between continuous variables were assessed using Spearman's (ρ) or Peason's (r) correlation coefficients, depending on distribution.

### Ethics statement

The study was approved by the Ethics Committees of the Malaysian Ministry of Health and Menzies School of Health Research. Informed written consent was provided by all participating adults, and by the parent or guardian of any participant aged <18 years.

## Supporting Information

S1 Text **In vitro production of PvLDH (S1A Fig.) and percentage of schizonts (S1B Fig.), and percentage of rings, trophozoites and schizonts (S1 Table).**
(DOCX)
