# Supplementary material for: Parasite Biomass-Related Inflammation, Endothelial Activation, Microvascular Dysfunction and Disease Severity in Vivax Malaria
Source: PLoS Pathog. 2015 Jan 8;11(1):e1004558. doi: 10.1371/journal.ppat.1004558 (PMC4287532; doi:10.1371/journal.ppat.1004558)
Supplement: S1 Text — In vitro production of PvLDH (S1A Fig.) and percentage of schizonts (S1B Fig.), and percentage of rings, trophozoites and schizonts (S1 Table). (DOCX) [file ppat.1004558.s001.docx]

**Text S1.** In vitro production of PvLDH (**Figure S1.A**) and percentage of schizonts (**Figure S1.B**), and percentage of rings, trophozoites and schizonts (**Table S1**).

**Figure S1.**

**A.**

**B.**

**Table S1.**

|  | **Culture time (hours)** | | | | | | | | |
| --- | --- | --- | --- | --- | --- | --- | --- | --- | --- |
|  | 0 | 6 | 10 | 12 | 24 | 30 | 34 | 48 | 54 |
| **A** | 98, 2, 0 | 97, 3, 0 |  | 97, 3, 0 | 76, 23, 1 | 12, 88, 0 |  | 0, 65, 35 | 0, 46, 54 |
| **B** | 98, 2, 0 |  |  |  | 64, 36, 0 |  |  | 19, 59, 22 |  |
| **C** | 65, 35, 0 | 70, 30, 0 |  | 67, 33, 0 | 42, 52, 6 | 15, 75, 10 |  | 43, 29, 28 | 16, 48, 36 |
| **D** | 98, 2, 0 |  | 97, 3, 0 |  | 48, 52, 0 |  | 46, 54, 0 | 15, 55, 30 | 46, 44, 10 |

Note: Numbers are % rings, % trophozoites and % schizont
